# Accuracy and uncertainty analysis of reduced time point imaging effect on time-integrated activity for ^177^Lu-DOTATATE PRRT in patients and clinically realistic simulations

**DOI:** 10.1186/s13550-023-01007-z

**Published:** 2023-06-12

**Authors:** Avery B. Peterson, David M. Mirando, Yuni K. Dewaraja

**Affiliations:** 1 Department of Radiology, University of Michigan, 1301 Catherine, 2276 Medical Science I/5610, Ann Arbor, MI 48109 USA; 2 Department of Radiation Oncology, Wayne State University, Detroit, MI USA; 3 MIM Software Inc, Cleveland, OH USA

**Keywords:** ^177^Lu, Radionuclide therapy, Dosimetry, Single time point, Reduced time point

## Abstract

**Background:**

Dosimetry promises many advantages for radiopharmaceutical therapies but repeat post-therapy imaging for dosimetry can burden both patients and clinics. Recent applications of reduced time point imaging for time-integrated activity (TIA) determination for internal dosimetry following ^177^Lu-DOTATATE peptide receptor radionuclide therapy have shown promising results that allow for the simplification of patient-specific dosimetry. However, factors such as scheduling can lead to sub-optimal imaging time points, but the resulting impact on dosimetry accuracy is still under investigation. We use four-time point ^177^Lu SPECT/CT data for a cohort of patients treated at our clinic to perform a comprehensive analysis of the error and variability in time-integrated activity when reduced time point methods with various combinations of sampling points are employed.

**Methods:**

The study includes 28 patients with gastroenteropancreatic neuroendocrine tumors who underwent post-therapy SPECT/CT imaging at approximately 4, 24, 96, and 168 h post-therapy (p.t.) following the first cycle of ^177^Lu-DOTATATE. The healthy liver, left/right kidney, spleen and up to 5 index tumors were delineated for each patient. Time-activity curves were fit with either monoexponential or biexponential functions for each structure, based on the Akaike information criterion. This fitting was performed using all 4 time points as a reference and various combinations of 2 and 3 time points to determine optimal imaging schedules and associated errors. 2 commonly used methods of single time point (STP) TIA estimation are also evaluated. A simulation study was also performed with data generated by sampling curve fit parameters from log-normal distributions derived from the clinical data and adding realistic measurement noise to sampled activities. For both clinical and simulation studies, error and variability in TIA estimates were estimated with various sampling schedules.

**Results:**

The optimal post-therapy imaging time period for STP estimates of TIA was found to be 3–5 days (71–126 h) p.t. for tumor and organs, with one exception of 6–8 days (144–194 h) p.t. for spleen with one STP approach. At the optimal time point, STP estimates give mean percent errors (MPE) within ± 5% and SD < 9% across all structures with largest magnitude error for kidney TIA (MPE = − 4.1%) and highest variability also for kidney TIA (SD = 8.4%). The optimal sampling schedule for 2TP estimates of TIA is 1–2 days (21–52 h) p.t. followed by 3–5 days (71–126 h) p.t. for kidney, tumor, and spleen. Using the optimal sampling schedule, the largest magnitude MPE for 2TP estimates is 1.2% for spleen and highest variability is in tumor with SD = 5.8%. The optimal sampling schedule for 3TP estimates of TIA is 1–2 days (21–52 h) p.t. followed by 3–5 days (71–126 h) p.t. and 6–8 days (144–194 h) p.t. for all structures. Using the optimal sampling schedule, the largest magnitude MPE for 3TP estimates is 2.5% for spleen and highest variability is in tumor with SD = 2.1%. Simulated patient results corroborate these findings with similar optimal sampling schedules and errors. Many sub-optimal reduced time point sampling schedules also exhibit low error and variability.

**Conclusions:**

We show that reduced time point methods can be used to achieve acceptable average TIA errors over a wide range of imaging time points and sampling schedules while maintaining low uncertainty. This information can improve the feasibility of dosimetry for ^177^Lu-DOTATATE and elucidate the uncertainty associated with non-ideal conditions.

**Supplementary Information:**

The online version contains supplementary material available at 10.1186/s13550-023-01007-z.

## Background

Treatment of gastroenteropancreatic neuroendocrine tumors with fixed activity ^177^Lu-DOTA-octreotide (^177^Lu-DOTATATE) has shown to increase overall survival and progression-free survival [1, 2]. Despite the promising results of standardized treatment, the need for patient-specific treatment options is indicated by the heterogeneity in pharmacokinetics, especially among tumors.

Dosimetry-guided peptide receptor radionuclide therapy (PRRT) can be used to maximize dose to tumors while ensuring that normal organs are spared from treatment-related toxicity. To perform patient-specific dosimetry accurately, generally multiple SPECT/CT acquisitions (minimum of 3 as recommend by [3]) are needed in the week following activity administration to quantify the radiopharmaceutical distribution and fit time-activity curves (TACs). This process imposes an imaging burden on not only the clinic but also the patient. In order to reduce this burden, there have been investigations of reduced and single-time point (STP) methods to estimate the time-integrated activity on a patient-specific basis while reducing the imaging burden. Hänscheid et al. [4] and Madsen et al. [5] have proposed two popular single time point methods that have been applied to ^177^Lu-DOTATATE imaging. The former relies on an approximation of a monoexponential function that is only valid at times near the effective half-life of the organ of interest. The latter assumes a population average effective half-life to produce an accurate estimate at a larger range of time points, but that still depends on each patient’s kinetics being similar to that of the population average. These methods have been evaluated [6–10], with Madsen being particularly robust over a wide range of assumed patient-specific effective half-lives and imaging time points; nevertheless, these studies cautioned against using STP methods [6, 7]. In addition, other reduced time point methods that employ 2 or 3 SPECT/CTs (2TP and 3TP) have been explored in ^177^Lu-DOTATATE [10–15] and other therapies [16–20].

These investigations into optimal imaging schedules for ^177^Lu-DOTATATE dosimetry with reduced time points have often shared common conclusions, primarily about the importance of including late time points [6, 11–13, 16] and the influence of early time points [6, 10, 11, 14, 15]. Investigations into STP methods also tend to recommend similar imaging times due to the necessity for the imaging time to be close to the effective half-life of the target organ. In order to balance the long half-life of tumors with the relatively short half-life of kidneys (a primary organ at risk in ^177^Lu-DOTATATE PRRT), STP imaging is often recommended at 72–96 h post-therapy (p.t.) [4, 8, 14].

Prior studies evaluating reduced time point imaging have typically focused only on evaluating performance for kidney [6, 9, 12–15] while generally being limited to only 3-time point reference data [6, 10, 14] or utilizing sub-optimal planar imaging [9, 13, 15]. Having access to 4-time point SPECT/CT imaging following ^177^Lu-DOTATATE PRRT for a cohort of patients and auto-segmentation tools to define multiple structures, we aim to perform a comprehensive evaluation of reduced time point methods through analysis of clinical patient data and simulated patient data with realistic measurement noise modeling. We investigate 1, 2, and 3 time point imaging, identify the optimal sampling schedules, and evaluate the error and variability in time-integrated activity (TIA) estimation with both optimal sampling and other schedules that are non-optimal but allow more flexibility to the clinic and patient.

## Methods

### Patients

Patients with gastroenteropancreatic neuroendocrine tumors who received at least one cycle of ^177^Lu-DOTATATE PRRT at the University of Michigan Hospital and volunteered for post-therapy SPECT/CT imaging as part of an ongoing IRB-approved research protocol were eligible for this study. A total of 28 patients met these criteria and underwent 4-time point SPECT/CT imaging for subsequent dosimetry. Patient characteristics are summarized in Table 1.

**Table 1:** Patient characteristics summary. Data is given as median (range) for numerical variables and N (%) for categorical variables

Characteristic	Data
Sex (Female/Male)	11/17 (39%/61%)
Age (y)	66 (38–76)
Weight (kg)	88.1 (51.7–129.3)
Diabetes (No/Yes)	22/6 (79%/21%)
Hypertension (No/Yes)	10/18 (36%/64%)
Grade (G1/G2/G3)*	8/15/1 (29%/54%/4%)
ECOG score	1 (0–2)
Number of prior systemic therapies	0 (0–4)
Number of prior liver-directed therapies	0 (0–2)
Estimated glomerular filtration rate (mL/min)	84.6 (31.8–102.7)
Administered ^177^Lu-DOTATATE (GBq)	7.3 (3.7–7.5)

^*^Tumor grade was unavailable for 4 (14%) of patients

### ^177^Lu SPECT/CT Imaging

The post-therapy imaging process has previously been described in detail [21] and is summarized here. ^177^Lu SPECT/CT imaging was performed on a Siemens Intevo at approximately 4, 24, 96, and 168 h p.t. following ^177^Lu-DOTATATE administration. Single-bed SPECT acquisitions of the abdomen were 25 min (2 heads, 120 views, 25 s per view) and used manufacturer-recommended settings (20% acquisition window at 208 keV with 10% scatter windows). Data were reconstructed with Siemens xSPECT Quant using ordered subset conjugate gradient maximization (48 iterations, 1 subset) with resolution recovery, outputting images in units of activity (Bq/mL) with matrix size 256 × 256 × 199 (1.953 mm^3^). No post-filtering was applied. Partial volume correction was applied to delineated structures using volume-based recovery coefficients determined from previous phantom experiments [21]. CT acquisitions were performed at 120 kVp and 80 mAs at one TP (“reference SPECT/CT”) and 15 mAs at all other TPs. The reconstructed matrix size was 512 × 512 × 130 (0.97 mm × 0.97 mm × 3 mm).

### Volumes of interest delineation

For each patient, the largest tumors (up to 5 per patient) were outlined by a radiologist using baseline imaging (CT and MR) and rigidly transferred to post-therapy ^177^Lu SPECT/CT images. Tumors with volume < 2 mL or located in bone were not considered for analysis due to uncertainties associated with segmentation and poor SPECT resolution.

The following normal structures were also delineated and considered for analysis: healthy liver, left/right kidney, and spleen. Total liver and left/right kidneys were segmented on the CT of the reference ^177^Lu-DOTATATE SPECT/CT using an automatic deep-learning-based model (MIM Software). Manual slice-by-slice spleen contours and, as needed, manual fine-tuning of total liver and left/right kidney deep-learning-based contours were performed by a medical physicist. Delineated tumor volumes were removed from the total liver contour to give the “healthy liver.”

### Clinical data

#### Reference time-activity curve fitting and integration

The SPECT images were co-registered with a contour-intensity-based SPECT-SPECT registration (MIM Software) [21] and the mean activity in each of the segmented structures as a function of time was fit to either a monoexponential curve of the form $C\times exp(-\lambda t)$ or a biexponential curve of the form $C\times \left(exp(-{\lambda }_{1}t)-exp(-{\lambda }_{2}t)\right)$ based on the Akaike information criterion as proposed by Sarrut et al. [22]. Exponential functions with more terms (e.g. 4-parameter biexponential) were not considered since our data was limited to 4-time points and functions fit with the number of data points ≤ number of free parameters are often underconstrained in that there are many combinations of parameters that fit the data well [23]. The analytic TIA for the exponential fit functions calculated for each structure for each patient in the clinical dataset was considered as the reference for comparison.

#### Reduced time point fitting

Patient time-activity data was grouped into 4 time periods corresponding approximately to scans performed on days 0 (${t}_{D0}$: 3-5 h), 1–2 (${t}_{D1-2}$: 21–52 h), 3–5 (${t}_{D3-5}$: 71–126 h), and 6–8 (${t}_{D6-8}$: 144–194 h) following treatment. For each structure of each patient, the activities from every possible combination of 2 and 3 time points were fit to a monoexponential function and compared to the reference TIA. This led to 6 combinations for 2 and 4 combinations for 3 time points. Note that not all patients had scans that matched one-to-one with the designated time periods. We also evaluate the single time point calculation methods of Hänscheid et al. [4] and Madsen et al. [5], henceforth simply referred to as the Hänscheid and Madsen method, respectively.

### Simulated data

Due to the relatively small patient data set (N = 28) and the discrete nature of patient time-activity data, we generated additional, clinically realistic time-activity curves by simulation. Simulation was performed in 3 parts as described in the following paragraphs: time-activity curve generation, activity sampling, and adding noise.

#### Time-activity curve generation

250 simulated TACs were generated for each evaluated structure (healthy liver, kidney, spleen, and tumor). The distribution of monoexponential and biexponential fits from the clinical dataset was maintained. Within each fit type, a lognormal distribution [8] was used for each exponential fit parameter ($C$ and $\lambda$ or $C$, ${\lambda }_{1}$, and ${\lambda }_{2}$ of the clinical dataset) of the form:

$$F(x) = \frac{1}{sx\sqrt{2\pi }}\times exp\left(-\frac{\left(\frac{x}{\mu }\right) }{2{s}^{2}}\right)$$

where $x$ is the parameter value, $s$ is the standard deviation of the natural logarithm of the clinical data for that parameter, and $\mu$ is the exponential of the mean of the natural logarithm of the clinical data for that parameter. To ensure realistic TACs and capture a wide range of clinical possibilities, the fitted lognormal distribution for each sampled parameter was further restricted to the minimum and maximum within the dataset; or, for the case of $\lambda$ for monoexponentials and ${\lambda }_{1}$ for biexponentials, if a smaller minimum or larger maximum for effective half-life was reported in the studies summarized by Hou et al. [8], then that value was used as a cutoff instead.

#### Sampling activity from simulated curve fits

For each simulated curve, activity was sampled at 1-h intervals from 1 to 240 h post-injection for testing STP methods. For 2TP fitting, activity was sampled at 4 h intervals and similarly; for 3 TP fitting, sampling was performed at 4 h intervals but with restrictions that prevented any two time points from being on the “same day” (within-12 h) or “overnight” (assuming therapeutic injection occurred at the beginning of the day at time 0 h, starting 4 h after injection, the only valid sampling times are at the beginning (24 h, 48 h, 72 h, …), middle (4 h, 28 h, 52 h, …), and end (8 h, 32 h, 56 h, …) of each day). This resulted in 240 possible times for the STP method, 1770 combinations for the 2TP method, and 3294 combinations for the 3TP method.

#### Measurement noise

SPECT imaging is affected by measurement noise, especially in low-uptake regions or at late time points. Thus, it is important to account for noise when simulating TACs. To estimate measurement noise to include in the virtual time-activity data, we performed repeat imaging (4 times) of a ^177^Lu phantom to determine the variability in counts. This process was repeated 10 times with varying acquisition times to imitate the decreased count-rate over approximately 10 days that would be expected due to physical and biological decay, although all measurements were performed on the same day. 7 phantom inserts of various shapes and sizes in an anthropomorphic abdominal phantom were filled with 4 different activity concentrations (Additional file 1: Fig. S1). The target activity concentrations were defined based on average values over multiple patients on day 0 SPECT imaging for kidney, healthy liver, tumors, and remainder of body (background). The healthy liver was filled with 258 kBq/mL; 2 uniform spheres and 1 uniform ellipsoid were filled with 1682 kBq/mL; 1 uniform ellipsoid and one sphere with a cold center were filled with 421 kBq/mL; and the background was filled with 39 kBq/mL. The filled activities were all within 10% of the target activities. Activity quantification of the repeat imaging with each scan length was used to compute a relative standard deviation for each object and scan length. The “effective activity” of each region was also found for each object and scan length which corresponds to the activity that would have resulted in the same number of decays if the scan length would have been 25 min (similar to a patient scan). The relative standard deviations and effective activities were used to fit a power law that models measurement noise as a function of activity.

The power law function was then used to add measurement noise at each sampled activity value by providing the standard deviation of a normal distribution about each sampled point along the simulated time-activity curves.

### Optimal time points and error determination

For each structure of each clinical and simulated patient, TIA was computed using reduced time point methods as described above for comparison to the relevant reference TIA. For each time point combination (sampling schedule), the accuracy of the STP and reduced TP imaging methods was evaluated using root mean square error (RMSE), mean percent error (MPE) with associated standard deviation (SD), and mean absolute percent error (MAPE). RMSE was used to compare sampling schedules within a particular structure and reduced time point method and was calculated as:

$$RMSE=\sqrt{\frac{{\sum }_{i=1}^{n}{\left(TI{A}_{i,pred}-TI{A}_{i,ref}\right)}^{2}}{n}}$$

where, $pred$ refers to the TIA calculated with reduced TP methods while $ref$ denotes the reference TIA. MPE indicates the average percent difference between TIA estimated with reduced TPs and reference TIA for a particular structure, reduced time point method, and sampling schedule but may hide large positive and negative errors if they are on average unbiased:

$$MPE=\frac{1}{n}{\sum }_{i=1}^{n}\frac{TI{A}_{i,pred}-TI{A}_{i,ref}}{TI{A}_{i,ref}}\times 100\%$$

MAPE is the average value of the absolute percent difference between TIA estimated with reduced TPs and reference TIA and is influenced in the same direction by both positive and negative errors:

$$MAPE=\frac{1}{n}{\sum }_{i=1}^{n}\left|\frac{TI{A}_{i,pred}-TI{A}_{i,ref}}{TI{A}_{i,ref}}\right|\times 100\%$$

The optimal sampling schedule for each structure was defined as the sampling schedule with the lowest RMSE across all patients (clinical or simulated).

## Results

### Clinical data

#### STP methods

**Fig. 1:**
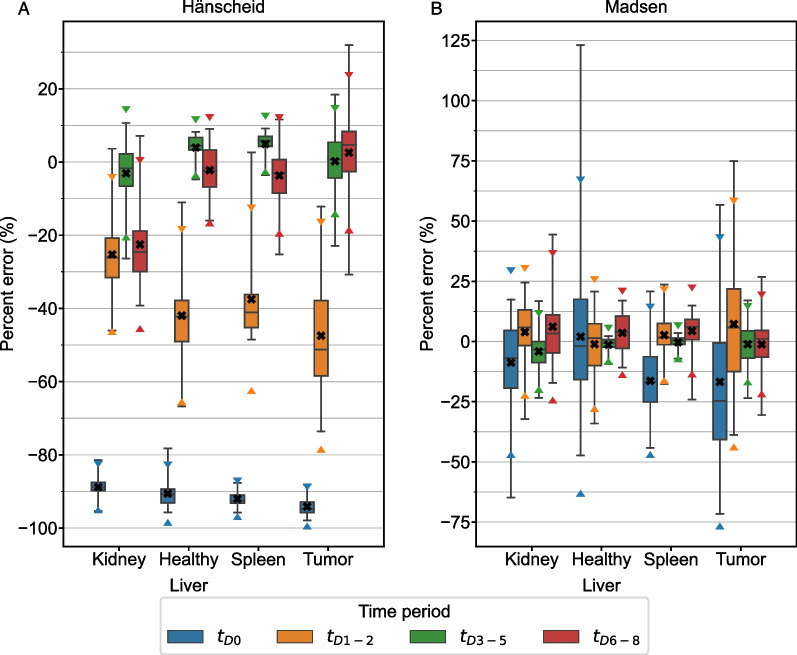
Percent error distribution for Hänscheid (**A**) and Madsen (**B**) STP methods for the clinical patient data grouped into 4 time periods. Box plots indicate minimum, maximum, median, 25th, and 75th percentile cutoffs. The black “X” identifies the mean value for each time period with triangle markers indicating the 95% confidence interval

The STP Hänscheid and Madsen methods were evaluated for patient time-activity data at the 4 defined time periods post-radiopharmaceutical injection. The percent error distribution of the STP predictions is presented in Fig. 1 for each structure. ${t}_{D3-5}$ was the time period with lowest RMSE across both STP methods for all structures except ${t}_{D6-8}$ slightly outperformed (RMSE 7% lower) ${t}_{D3-5}$ when the Hänscheid method was applied to the spleen. The optimal time period and various measures of error are summarized for both methods and all four structures in Table 2.

**Table 2:** Optimal sampling schedules (lowest RMSE across all sampling schedules) for each structure and reduced time point method (Hänscheid, Madsen, 2TP, and 3TP) using measured clinical patient data with RMSE, MPE (SD), and MAPE for that sampling schedule

Struct	Method	Optimal TP	RMSE (MBq-h)	MPE (SD) [range] (%)	MAPE (%)
Kidney	Hänscheid	t_D3-5_	561	− 3.1 (8.4) [− 26.4–10.7]	6.6
Kidney	Madsen	t_D3-5_	603	− 4.1 (7.4) [− 23.4–16.7]	6.3
Kidney	2TP	t_D1-2_,t_D3-5_	254	1.0 (3.7) [− 7.2–19.3]	2.3
Kidney	3TP	t_D1-2_,t_D3-5_,t_D6-8_	219	2.2 (1.8) [− 4.8–5.9]	2.4
Liver	Hänscheid	t_D3-5_	5168	4.0 (3.4) [− 4.8–8.2]	4.8
Liver	Madsen	t_D3-5_	3122	− 1.3 (2.8) [− 9.3–2.2]	2.2
Liver	2TP	t_D3-5_,t_D6-8_	1482	0.6 (2.2) [− 8.0–4.0]	1.7
Liver	3TP	t_D1-2_,t_D3-5_,t_D6-8_	1367	1.2 (1.5) [− 4.3–4.3]	1.6
Spleen	Hänscheid	t_D6-8_	820	− 3.7 (7.6) [− 25.2–11.6]	6.2
Spleen	Madsen	t_D3-5_	270	− 0.3 (2.8) [− 7.2–3.4]	2.1
Spleen	2TP	t_D1-2_,t_D3-5_	333	1.2 (2.8) [− 3.5–10.5]	2.0
Spleen	3TP	t_D1-2_,t_D3-5_,t_D6-8_	487	2.5 (1.6) [0.2–7.2]	2.5
Tumor	Hänscheid	t_D3-5_	1629	0.2 (6.9) [− 22.9–18.4]	5.5
Tumor	Madsen	t_D3-5_	1061	− 1.1 (7.3) [− 23.5–17.1]	5.9
Tumor	2TP	t_D1-2_,t_D3-5_	558	0.9 (5.8) [− 17.7–20.6]	4.0
Tumor	3TP	t_D1-2_,t_D3-5_,t_D6-8_	424	2.1 (2.1) [− 6.6–14.2]	2.2

#### Multi-time point

**Fig. 2:**
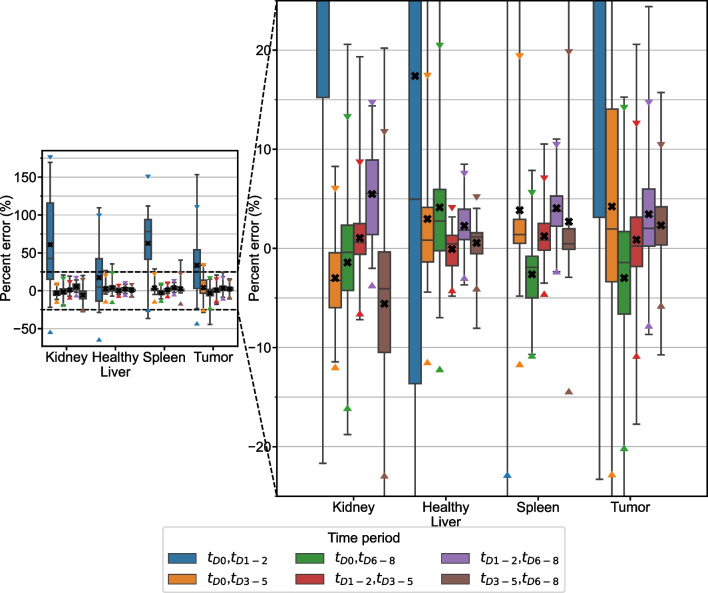
Percent error distribution for TIA estimates from 2TP fitting of the clinical patient data grouped into 4 time periods. Box plots indicate minimum, maximum, median, 25^th^, and 75^th^ percentile cutoffs. The black “X” identifies the mean value for each time period with triangle markers indicating the 95% confidence interval

**Fig. 3:**
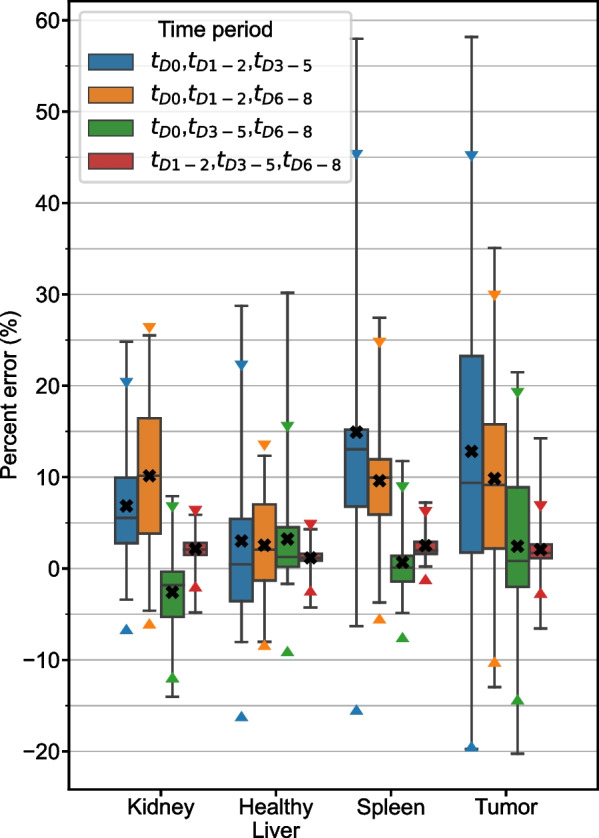
Percent error distribution for TIA estimates from 3TP fitting of the clinical patient data grouped into 4 time periods. Box plots indicate minimum, maximum, median, 25th, and 75th percentile cutoffs. The black “X” identifies the mean value for each time period with triangle markers indicating the 95% confidence interval

Results of fitting 2TP combinations of time periods with monoexponential functions are represented by boxplots in Fig. 2. The time period combinations with lowest RMSE were ${t}_{D1-2},{t}_{D3-5}$ for kidney, spleen, and tumor and ${t}_{D3-5}{,t}_{D6-8}$ for liver. These optimal schedules are presented in Table 2 alongside associated measures of error. MPE in TIA prediction for 3TP combinations are presented in Fig. 3 and the time period combination with lowest RMSE was ${t}_{D1-2},{t}_{D3-5},{t}_{D6-8}$ for all structures. Measures of error for this optimal sampling schedule are also presented in Table 2.

### Noise assessment in phantom

**Fig. 4:**
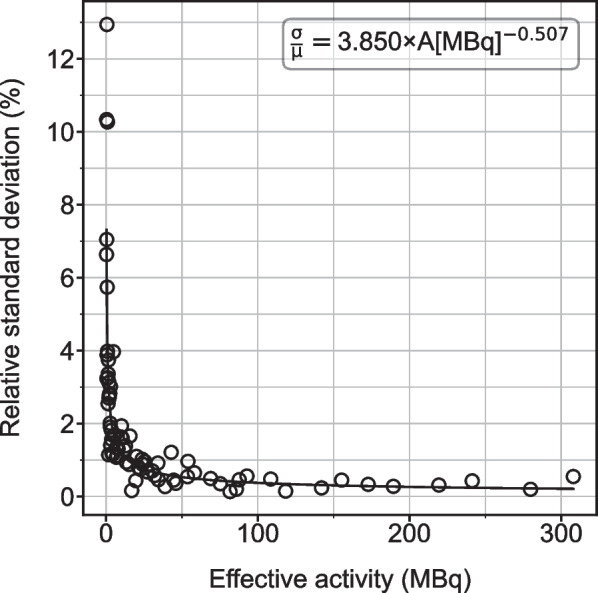
Relative standard deviation (%) plotted as a function of effective activity from phantom measurements to estimate noise. The fit line, as determined from ordinary least squares regression of a log–log transformation of this data, is plotted as a solid line. The fit equation is also given

The results of the phantom experiment are presented in Fig. 4 with the average effective activity across 4 samples plotted against the relative standard deviation of those samples. A log–log transformation of the data indicates that a power-law reasonably describes the noise as a function of effective activity. The coefficients of the power-law were determined using ordinary least squares regression on the log–log transformation of the data presented in Fig. 4.

### Simulated patients

**Fig. 5:**
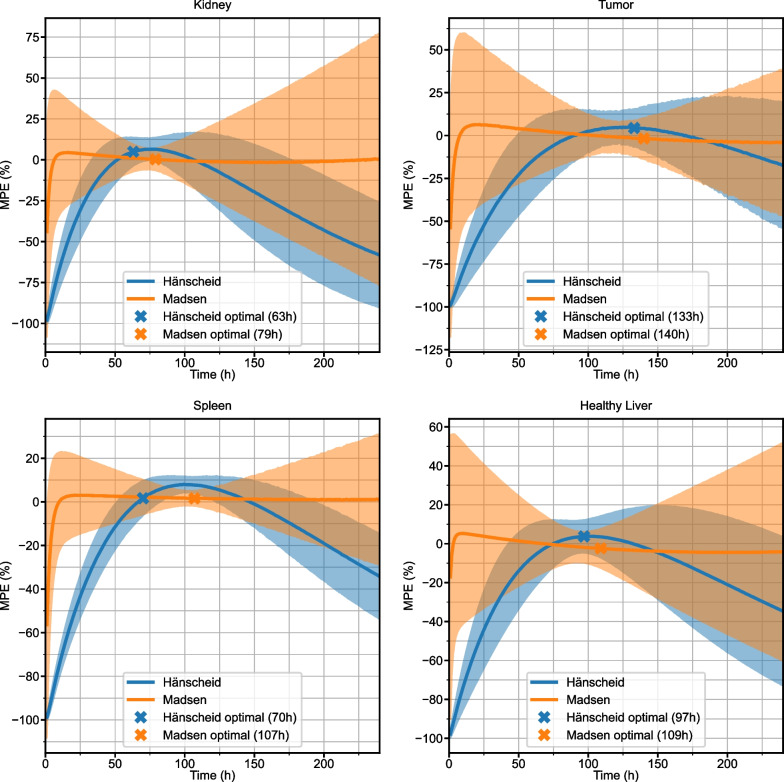
MPE with 95% confidence intervals for TIA estimates using the Hänscheid (blue) and Madsen (orange) STP methods as a function of sampling time for simulated time activity curves with added measurement noise. Indicated optimal time points correspond to the minimum RMSE

**Fig. 6:**
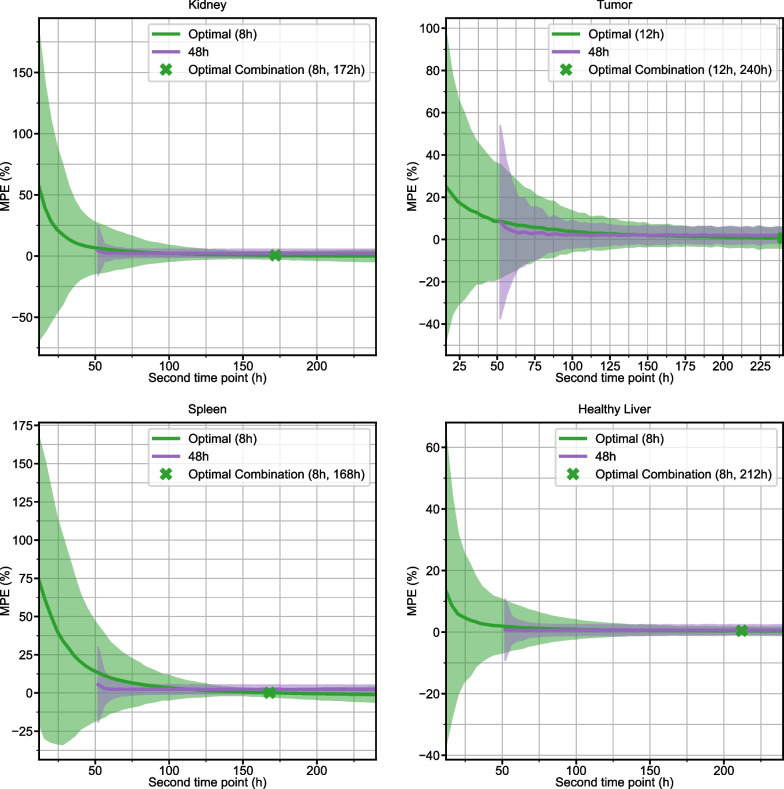
For each structure, the MPE with 95% confidence interval for TIA prediction of simulated 2TP combinations is presented for two different first time points: the optimal time point (green) and 48 h (purple). The optimal combination of first and second time point, based on minimum RMSE, is indicated by a green “X”

250 different simulated curves for each structure were generated with measurement noise added to the sampling points before refitting. MPE with 95% confidence interval for the Hänscheid and Madsen STP methods is plotted for each time point in Fig. 5. 2TP sampling schedules for two different first time points (optimal and 48 h) are plotted in Fig. 6. MPE and SD for all 2TP sampling schedules are given as 2D heatmaps in Additional file 1: Figs. S2–S5. These heatmaps indicate that there are many sampling schedules that exhibit MPE <  ± 5%, even with added measurement noise. Table 3 summarizes the RMSE, MPE (SD), and MAPE at the optimal time point combinations for STP, 2TP, and 3TP sampling methods. A tool that computes various error metrics for the requested non-optimal 1, 2, or 3 time point sampling schedule and provides a visualization of the error has been made available online [24].

**Table 3:** Optimal sampling schedules (lowest RMSE across all sampling schedules) for each structure and reduced time point method (Hänscheid, Madsen, 2TP, and 3TP) using simulated data with RMSE, MPE, and MAPE for that sampling schedule

Struct	Method	Optimal TP (h)	RMSE (MBq-h)	MPE (SD) [range] (%)	MAPE (%)
Kidney	Hänscheid	63	445	4.9 (4.4) [− 14.0–12.4]	6.0
Kidney	Madsen	79	228	0.3 (3.4) [− 18.1–6.2]	2.4
Kidney	2TP	8, 172	98	0.6 (1.4) [− 6.4–3.5]	1.3
Kidney	3TP	8, 148, 220	96	0.7 (1.3) [− 4.5–3.4]	1.2
Liver	Hänscheid	97	4035	3.7 (4.3) [− 23.5–8.7]	5.1
Liver	Madsen	109	3028	− 2.4 (5.1) [− 36.7–2.4]	3.0
Liver	2TP	8, 212	524	0.5 (0.6) [− 0.8–2.0]	0.6
Liver	3TP	8, 200, 224	525	0.4 (0.6) [− 0.9–2.1]	0.5
Spleen	Hänscheid	70	569	1.7 (4.0) [− 10.8–9.8]	3.7
Spleen	Madsen	107	302	1.6 (1.8) [− 5.5–5.9]	2.0
Spleen	2TP	8, 168	161	0.2 (1.3) [− 4.1–2.8]	1.0
Spleen	3TP	8, 144, 220	142	0.2 (1.2) [− 3.4–2.6]	1.0
Tumor	Hänscheid	133	1362	4.4 (5.6) [− 29.2–12.7]	6.2
Tumor	Madsen	140	646	− 1.8 (6.1) [− 36.3–7.4]	3.9
Tumor	2TP	12, 240	317	0.7 (2.4) [− 15.3–8.7]	1.8
Tumor	3TP	24, 224, 240	495	1.9 (1.6) [− 6.6–8.6]	2.1

## Discussion

We demonstrate that it is possible to obtain robust TIA estimates from reduced time point methods. For optimal sampling schedules, MPE remains within ± 5% for all reduced time point methods across all structures in both clinical patients and simulation (Table 2, 3). Variability at the optimal sampling schedules also remains low, with maximum SD of 8.4% across all structures and reduced time point methods. The single time point results agree well with the findings of Hänscheid et al. [4], Hou et al. [8], and Zhao et al. [14] that indicate 72–96 h p.t. as an optimal time frame for single time point estimation as it captures the kinetics of both the kidneys and tumor adequately. We note that the Hänscheid approximation is less robust than the Madsen method as the sampling time moves away from the population effective half-life with large negatively biased errors (Fig. 1, 5). However, while the Madsen approximation remains unbiased across a wide range of sampling times, variability increases quickly with distance from the optimal time (e.g. MAPE for Kidney is 2.4% at the optimal time of 79 h, but increases to 8.5% at 120 h and 13.9% at 150 h).

2 TP imaging methods are able to achieve high degrees of accuracy, particularly when early time points are coupled with late time points. Choosing two early (< 48 h p.t.) time points is not recommended as the error in TIA across all structures in the clinical and simulation datasets is large (~ 5–50%) with high variability, especially in tumor and kidney, whereas there are ample sampling schedules that have average error of <  ~ 2% (Figs. 2, 6, Additional file 1: Figs. S2–S5). Previous publications have also recommended against choosing imaging time points that are too early [6, 10, 11, 14, 15]. The slight overestimation that we observe in our data and that is present even in the optimal time point groupings is due to the monoexponential fits missing information about the uptake phase of the pharmaceutical that is measurable with early time point imaging. 3TP sampling schedules exhibited lower variability than 2TP, but improved MPE was generally only seen when one of the imaging time points was earlier than 48 h. In that case, the additional time point can capture information about the radiopharmaceutical uptake phase. Across the clinical and simulated data, however, 3 TP exhibited lower variability than 2 TP methods. As indicated in Table 2, dropping the early (t_D0_) time period from the 4-time point reference data results in the optimal 3 TP clinical sampling schedule with MAPE ranging from 2.1 to 2.5% depending on structure. Note that dropping the late (t_D6-8_) time period results in larger differences due to 3 TP monoexponential fitting with MAPE ranging from 6.7%-15.6% (Fig. 3).

The simulation results corroborate the results derived from the clinical patient data. The effect of measurement noise is negligible in most cases with the notable exception of large errors (> > 100%) and variability (> > 100%) associated with choosing 2 late time points that are too close to each other (Additional file 1: Figs. S2–S5). This effect is particularly important in low activity structures (e.g. small, low-uptake tumors) with relatively large measurement noise at late time points that result in unrealistic monoexponential fits. These curves fit the noisy data well, but when extrapolated, drastically overestimate the TIA. It is also worth noting that the techniques we used to model clinically realistic simulation data could be applied as a framework for investigating error and variability in reduced time point imaging for other radionuclide therapies.

Gustafson et al. [7] looked at single time point estimates for a range of biological half-lives. They observed that there is an upper positive bound on the single time point estimate bias but no lower bound, thus indicating a risk of underestimating dose with STP. Our results provide further clinical evidence for this theoretical claim, reinforcing the need to carefully choose imaging time point if single time point methods are going to be employed.

Focusing on renal dosimetry in a large cohort, Sandström et al. [6] advised against using STP methods altogether and instead recommended using two imaging time points at a minimum. They also stressed the importance of the time points chosen and generally recommend one early and one late time point. Our data shows that if you are careful about the imaging time point, the Hänscheid and Madsen STP methods are robust with low errors (MPE within ± 5% and SD < 9%) across normal organs and tumors. However, individual structures may still exhibit relatively large absolute error up to 26.4% using STP methods according to our measured patient data (Table 1) and our comparatively small sample size may hide variability that is observed in the larger sample analyzed by Sandström et al. Additionally, in the case of 2 TP methods, we observe that picking a first time point that is too early can lead to over and underestimation of TIA depending on the choice of second time point. Nonetheless, the variation in 2 TP TIA estimates using sub-optimal sampling schedules is often similar to the variation in STP estimates using optimal sampling schedules (Fig. 2). For example, the SD of the 2 TP estimates using the non-optimal time points ${t}_{D0}$ and ${t}_{D6-8}$ for the clinical kidney data is 7.3%, which is lower than the SD of either the Hänscheid or Madsen STP estimates (8.4% and 7.4%) at the optimal time period ${t}_{D3-5}$. This is consistent with the recommendation for two imaging time points from Sandström et al.

Our study provides a comprehensive overview of TIA error and variability as a result of using different reduced and single time point fitting methods to patient data and simulated time-activity curves. However, our current study possesses some limitations. While we expanded our analysis to include multiple normal organs by exploiting recently available auto-segmentation tools, bone marrow was not included as it is a complex structure that is not easily defined. Furthermore, we observed that time-activity in regions of marrow uptake were not well-fit by the 2 or 3 parameter exponential models that we used as the reference in this work considering that we have only 4 sampling points. Analysis of reduced time point methods for bone marrow will be undertaken in the future as we are in the process of developing tools for bone marrow dosimetry [25]. We are also limited in our clinical data by a small sample size of 28 patients with full 4-time point imaging. Our simulated data is similarly limited because the simulated time-activity curves informed by this limited sample size (although cutoffs for effective half-life incorporated values from other cohorts). Furthermore, our simulated curve fit parameters are assumed to follow log-normal distributions based on observations from other groups [8] and supported by KS tests but the true distribution is not known a priori. The reference clinical curve fits were based on 4-time point fitting of measured time-activity data. We chose to allow only 2 and 3 parameter exponential fits to this data because 4 parameter biexponentials were underconstrained for the 4-time point data, but organs of interest can exhibit a 2-phase clearance pattern that is not accurately captured by mono or bi-exponential fits. Simulations using physiologically-based pharmacokinetic models may provide more realistic curves that are not bound to monoexponential or biexponential functional forms, but are affected by uncertainty in the estimates of the physiological parameters [16–18, 20, 26]. It is also worth noting that there are other methods of STP and reduced time point dosimetry such as those that employ non-linear mixed models [27, 28] or Jackson et al. [29] which uses historical time-activity curves normalized to a single imaging time to estimate the mean and range of TIA, while we focus on 2 of the more common and simple implementations of STP dosimetry.

## Conclusions

We show that reduced time point methods can be used in ^177^Lu-DOTATATE PRRT to achieve acceptable average TIA errors for both tumor and normal organs over a wide range of imaging time points and sampling schedules for 1, 2, and 3 TP imaging regiments with low uncertainty. Provided clinics avoid imaging at two early time points (< 48 h p.t.), even when accounting for measurement noise, performing 2TP imaging can provided TIA estimates with average error and standard deviation less than 5% of the reference TIA for tumor and kidney. 3TP imaging provides similar performance but with generally lower variability. The 2 common STP methods investigated exhibit slightly higher average error and variability but still show MPE within ± 5% and SD < 9% for tumors and organs at optimal time points. STP imaging at time points much different from the optimal increases error and/or variability. Using reduced time point imaging saves time for the clinic and patient with only minor tradeoffs for reduced TIA accuracy, thus making patient-specific dosimetry for ^177^Lu-DOTATATE PRRT more accessible.

## Supplementary Information

**Additional file 1**. Description: Document containing Figs. S1–S5 referred to in the manuscript. Figures are labeled with descriptive captions within the document.

## Data Availability

Some of the datasets generated and analyzed during the current study and an accompanying Python tool are available online at, https://github.com/averybpeterson/reduced-tp-error-checker, and an archived version can be found at, https://doi.org/10.5281/zenodo.7843928 [24]. The remaining data is available from the corresponding author on reasonable request.

## Abbreviations

CT: Computed tomography
MAPE: Mean absolute percent error
MPE: Mean percent error
MR: Magnetic resonance
PRRT: Peptide receptor radionuclide therapy
p.t.: Post-therapy
RMSE: Root mean square error
SPECT: Single photon emission computed tomography
STP: Single time point
TAC: Time-activity curve
TIA: Time-integrated activity
TP: Time point
