# Supplementary material for: Accuracy and uncertainty analysis of reduced time point imaging effect on time-integrated activity for 177Lu-DOTATATE PRRT in patients and clinically realistic simulations
Source: EJNMMI Res. 2023 Jun 12;13:57. doi: 10.1186/s13550-023-01007-z (PMC10260735; doi:10.1186/s13550-023-01007-z)
Supplement: Supplementary file 1 — Additional file 1. Description: Document containing Figs. S1–S5 referred to in the manuscript. Figures are labeled with descriptive captions within the document. [file 13550_2023_1007_MOESM1_ESM.pdf]

SUPPLEMENTAL DATA

Contour Name	Volume (ml)	Activity concentration at filling (kBq/mL)
Background	9072.1	39
Healthy Liver	1137.4	258
Shell Sphere	118.2	421 (outer)<br>0 (inner)
Large Sphere	65.3	1682
Small Sphere	4.1	1682
Large Ellipsoid	28.5	1682
Small Ellipsoid	10.5	421

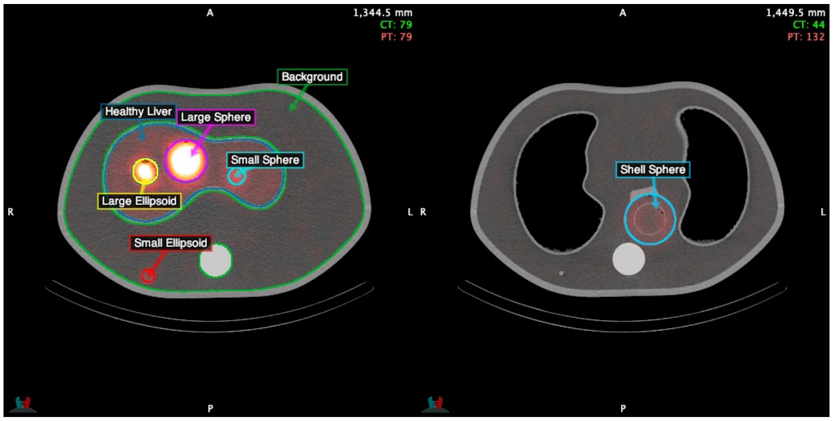

Supplemental Figure 1. Table indicating volume and shape of compartments used for measurement noise phantom experiment. Each structure outline is also shown on axial slices of the ^{177}Lu-DOTATATE SPECT/CTs taken of the phantom.

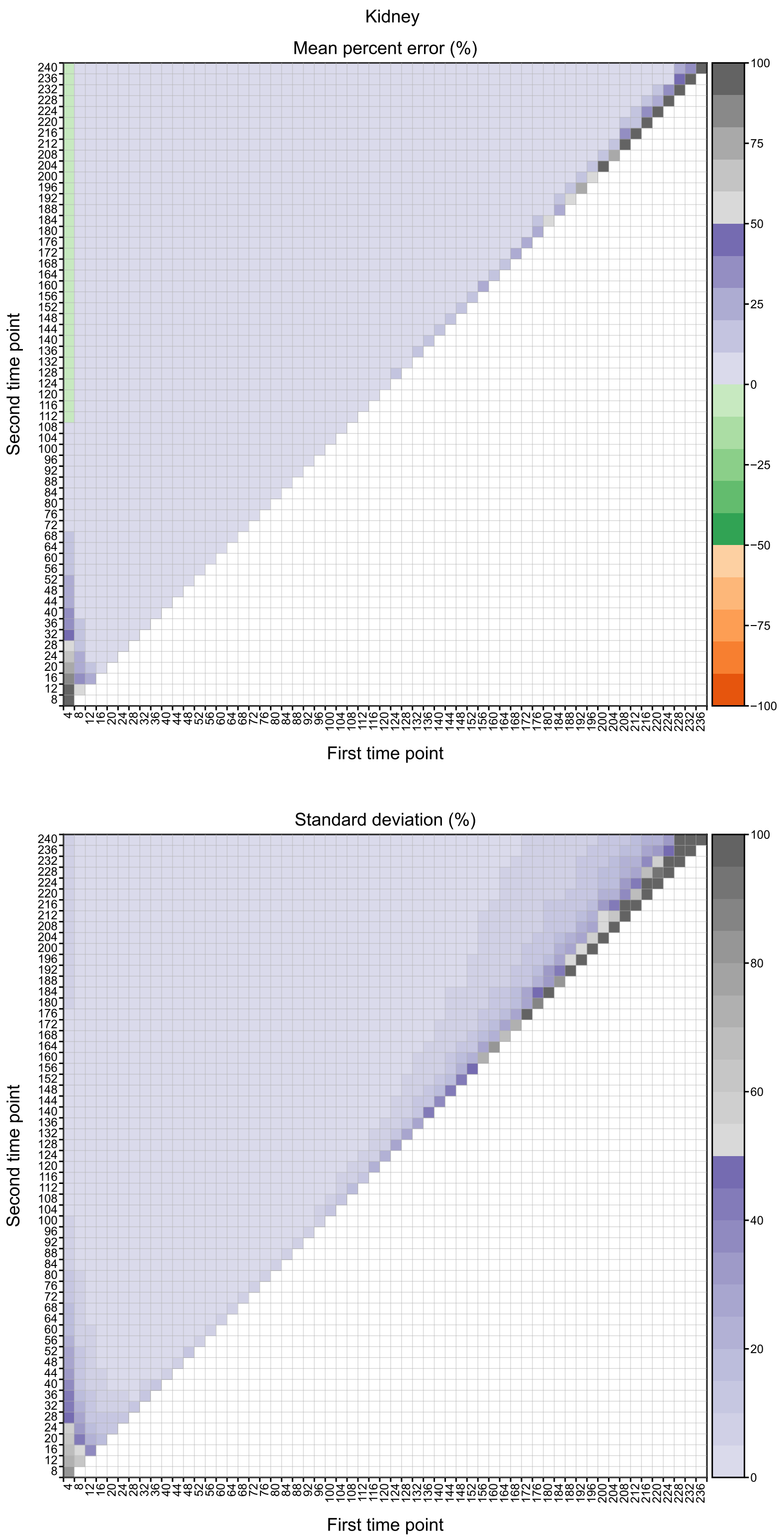

Supplemental Figure 2. Kidney A) mean percent error and B) standard deviation for all 1770 2TP combinations.

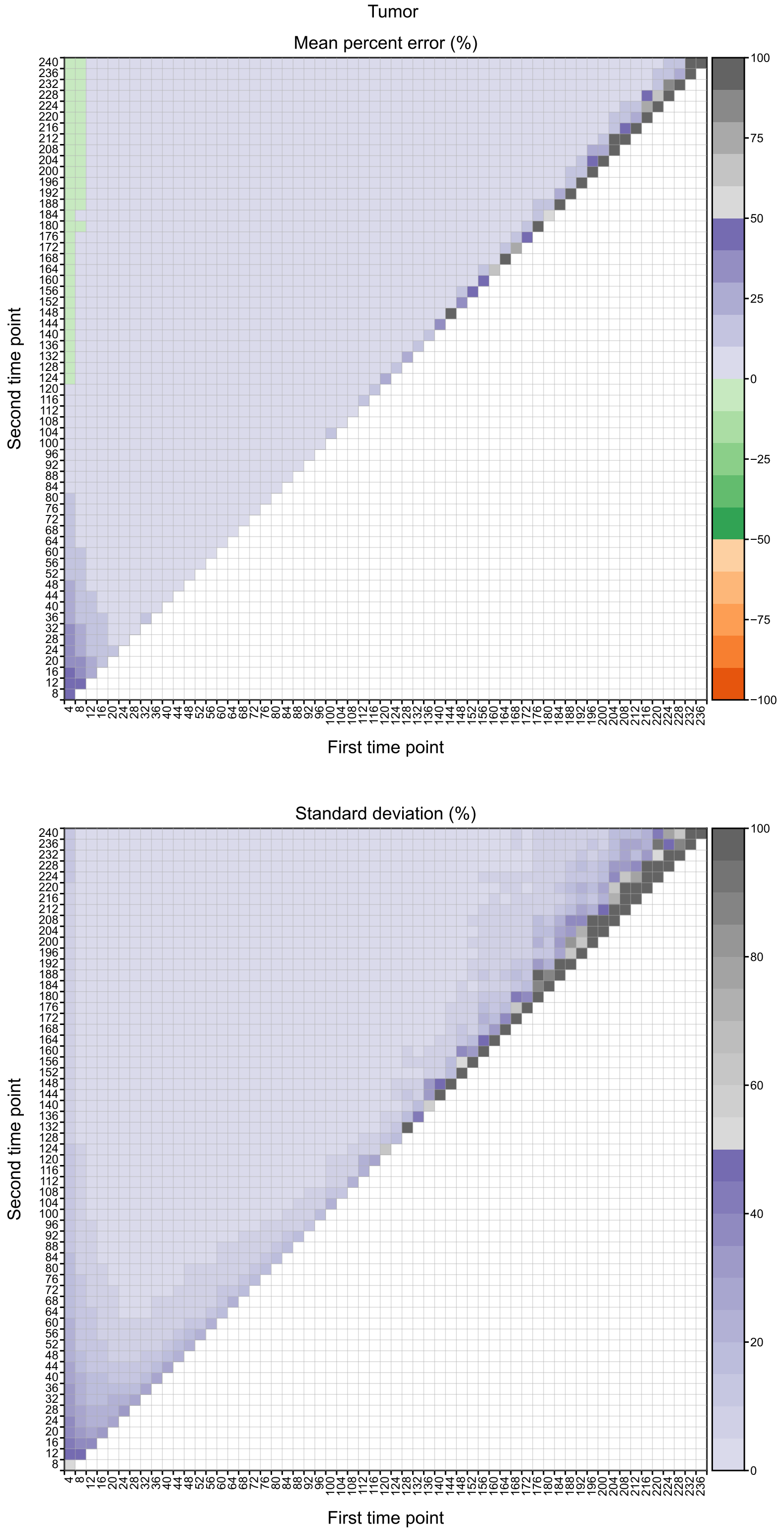

Supplemental Figure 3. Tumor A) mean percent error and B) standard deviation for all 1770 2TP combinations.

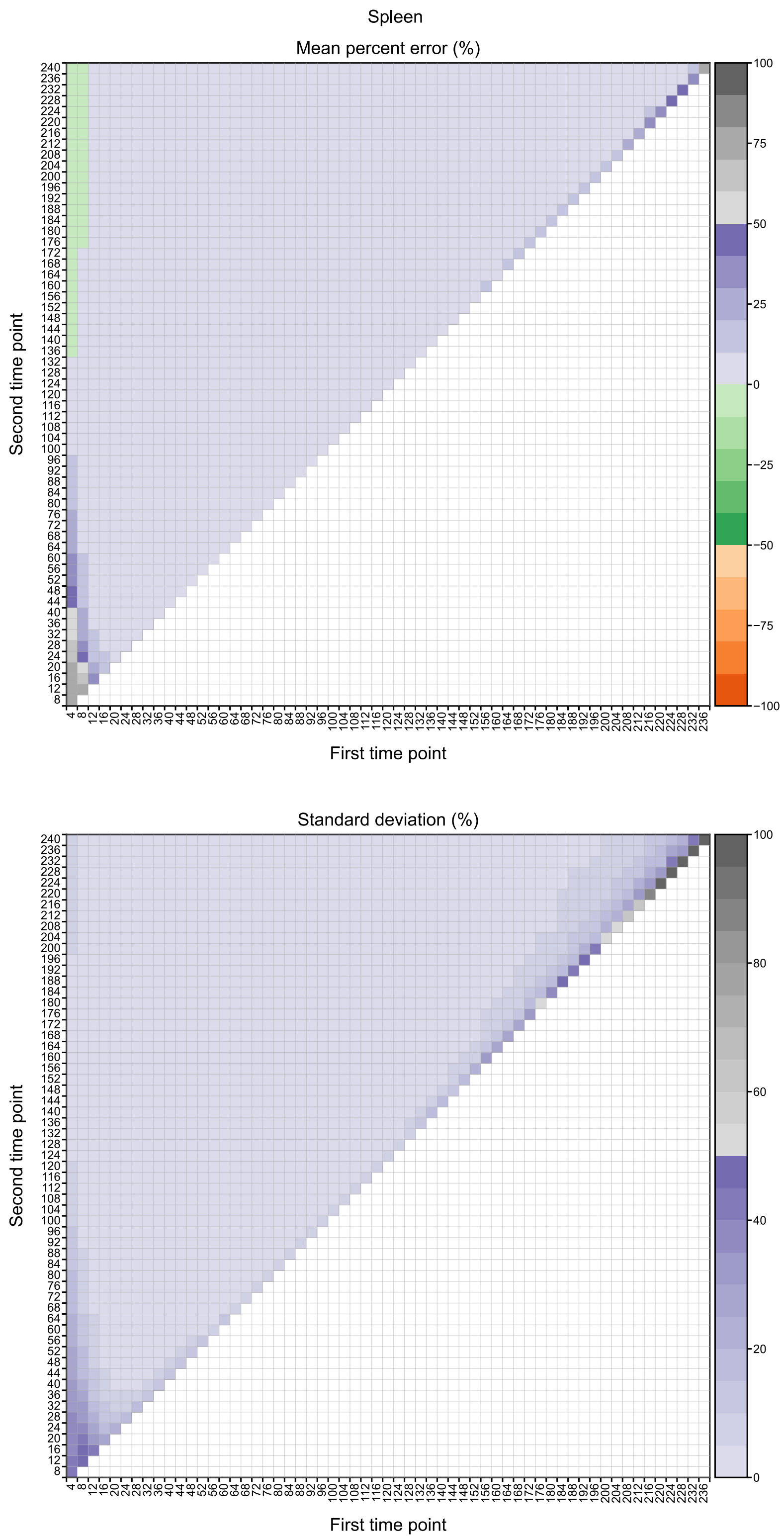

Supplemental Figure 4. Spleen A) mean percent error and B) standard deviation for all 1770 2TP combinations.

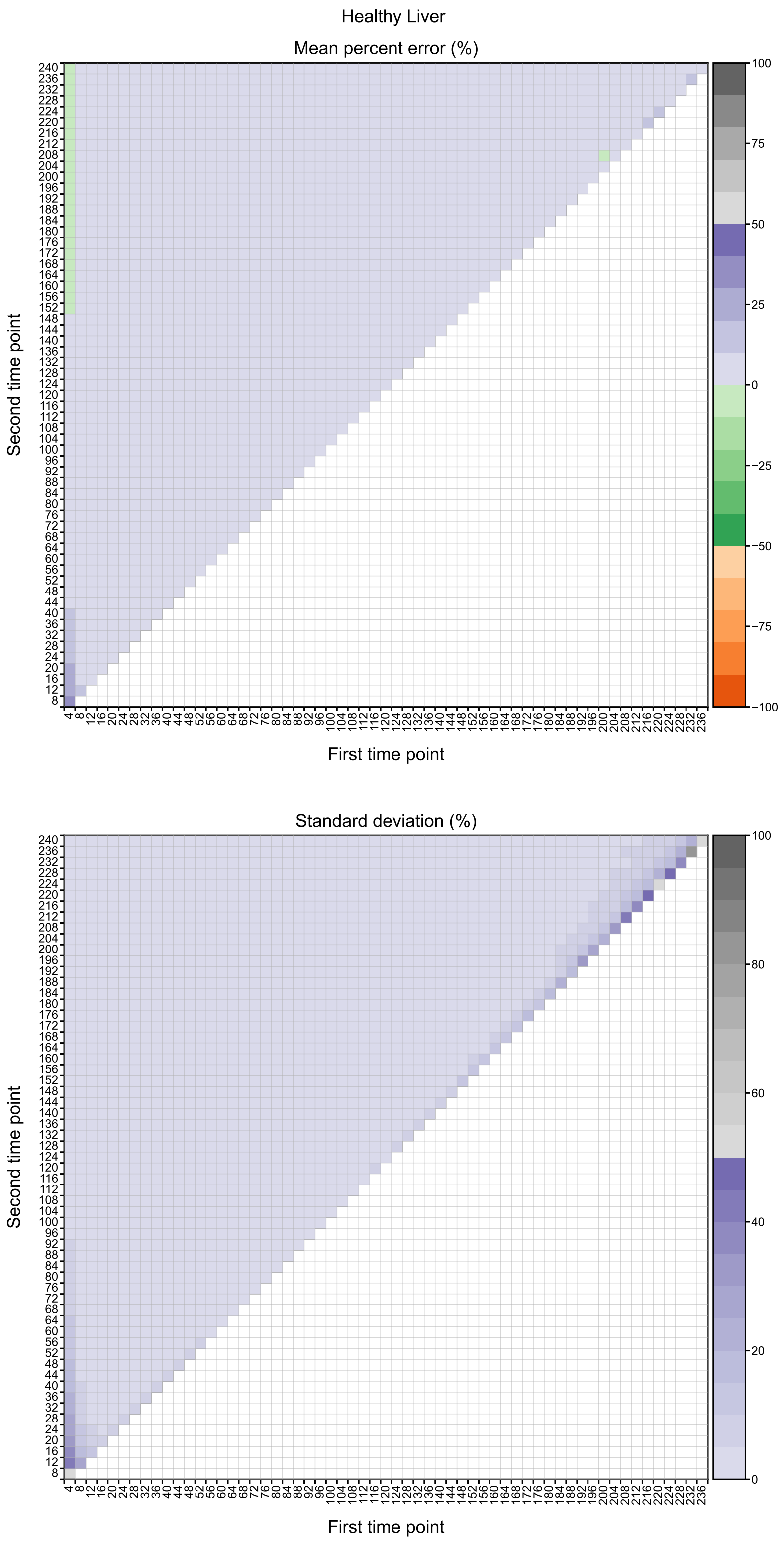

Supplemental Figure 5. Healthy liver A) mean percent error and B) standard deviation for all 1770 2TP combinations.
